# Malaria case in Madagascar, probable implication of a new vector, *Anopheles coustani*

**DOI:** 10.1186/s12936-015-1004-9

**Published:** 2015-12-01

**Authors:** Thiery N. J. J. Nepomichene, Etienne Tata, Sébastien Boyer

**Affiliations:** Unité d’Entomologie Médicale, Institut Pasteur de Madagascar, BP 1274, Ambatofotsikely, 101 Antananarivo, Madagascar; Ecole doctorale Sciences de la vie et de l’environnement, Université d’Antananarivo, Antananarivo, Madagascar

**Keywords:** Malaria, *Plasmodium falciparum*, *Plasmodium vivax*, *Anopheles arabiensis*, *Anopheles funestus*, *Anopheles mascarensis*, *Anopheles coustani*, Madagascar

## Abstract

**Background:**

Indoor spraying of insecticides and the use of insecticide-treated bed nets are key strategies for national malaria vector control in the central highlands of Madagascar. During the year 2013, malaria outbreaks were reported by the National Malaria Control Programme in the highlands, including the district of Ankazobe.

**Methods:**

Entomological trapping was carried out in April and May 2013 and in March 2014, using human landing catches, collection of mosquitoes resting in stables and in houses by oral aspirators, and Centers for Disease Control and Prevention light traps. Detection of *Plasmodium* in mosquitoes was carried out on head and thorax of anopheline females by ELISA, CSP and PCR (*Plasmodium falciparum*, *Plasmodium malariae*, *Plasmodium vivax*, or *Plasmodium ovale*). Human biting rate (HBR), sporozoite index and entomological infection rate (EIR) were calculated for *Anopheles funestus*, *Anopheles arabiensis,Anopheles mascarensis*, and *Anopheles coustani.*

**Results:**

In Ankazobe district, the presence of malaria vectors such as *An. funestus*, *An. arabiensis* and *An. mascarensis* was confirmed, and a new and abundant potential vector, *An. coustani* was detected. Indeed, one individual of *An. funestus* and two *An. coustani* were detected positive with *P. falciparum* while one *An. mascarensis* and four *An. coustani* were positive with *P. vivax*. For *An. coustani,* in March 2014, the EIR varied from 0.01 infectious bites/person/month (ipm) outdoors to 0.11 ipm indoors. For *An. funestus*, in April 2013, the EIR was 0.13 ipm. The highest HBR value was observed for *An. coustani*, 86.13 ipm outdoors. The highest sporozoite rate was also for *An. coustani*, 9.5 % of *An. coustani* caught in stable was sporozoite positive.

**Conclusion:**

The implication of *An. coustani* in malaria transmission was not previously mentioned in Madagascar. Its very high abundance and the detection of *Plasmodium* coupled with an opportunistic feeding behaviour in villages with malaria cases supports its role in malaria transmission in Madagascar.

## Background

In Central Highlands of Madagascar (CHM), an intensive campaign to eliminate malaria begun in 1949 by applying indoor residual spraying (IRS) with DDT [1]. The abandonment of IRS in 1979, the discontent with health structures with a slow erosion of health facilities combined with the absence of drug stock and medical staff absenteeism led to a malaria outbreak in 1986 [2–5]. In response, a vector control program was implemented with DDT in CHM from 1993 to 1998. From 1999, systematic IRS was replaced by selective operations in restricted areas 900 m above sea level (asl) in CHM. In 2005, carbamate insecticide replaced DDT.

*Anopheles funestus*, *Anopheles mascarensis*, *Anopheles gambiaes.s.*, *Anopheles arabiensis,* and *Anopheles merus* were considered primary vectors of malaria in Madagascar [6–9]. *Anopheles funestus* was considered the major vector of *Plasmodium falciparum* malaria in the CHM and *An. arabiensis* a secondary vector. Following the residual spraying from 1949 to 1979, *An. funestus* disappeared from most CHM villages [3, 5, 10]. Its re-invasion was mentioned in CHM in 1986 [2–5]; malaria outbreaks occurred with one specimen of *An. funestus* detected positive with *Plasmodium* despite a low abundance [5]. Other species such as *An. coustani, Anopheles squamosus/cydippis* were suspected to contribute to the epidemic [5]. However, to date, there have been no specific studies carried out on *An. coustani* and *An. squamosus/cydippis*.

Twenty-seven years after the last epidemic, a malaria outbreak resurged in the CHM in 2013. A high number of malaria cases were declared in Ankazobe district. The present work had the objective to identify the malaria vectors in CHM that could be responsible for the epidemic.

## Methods

### Study sites and period of capture

**Fig. 1:**
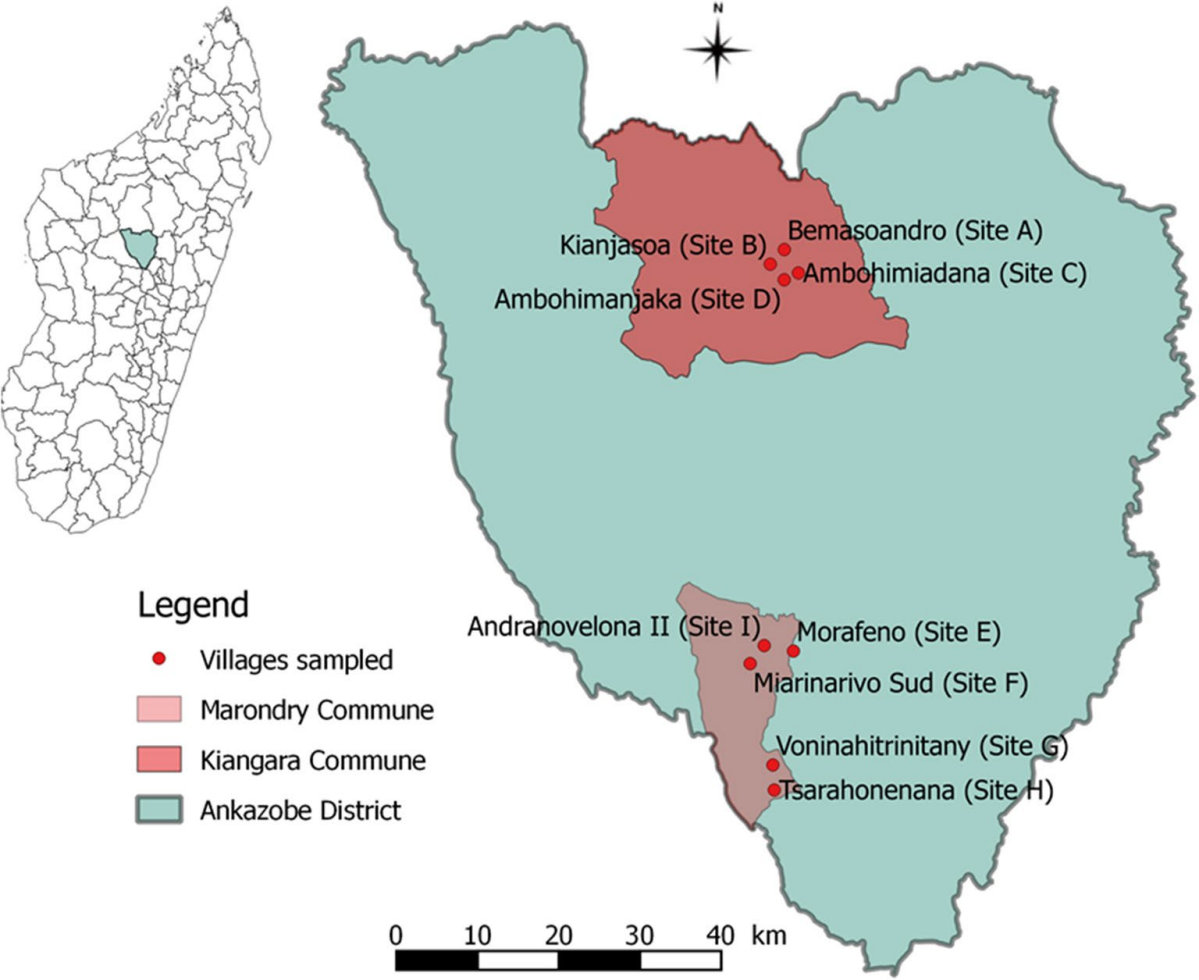
Study sites

Entomological surveys were conducted in two communes: Kiangara and Marondry within the Ankazobe district (Fig. 1). The site of Andranovelona II (Site I, S 18°23′25.2′′/EO 47°01′00.0′′) was sampled in April and May 2013 whereas the sites of Bemasoandro (Site A, 17°S 57′26; 47°EO 02′60), Kianjasoa (Site B, 17°S 58′25; 47°EO 01′80), Ambohimiadana (Site C, 17°S 58′59; 47° EO 03′26), Ambohimanjaka (Site D, 17°S 59′27; 47°EO 02′07), Morafeno (Site E, 18°S 24′13; 47°EO 03′03), Miarinarivo Sud (Site F, 18°S 26′05; 47°EO 00′02), Voninahitrinitany (Site G, 18°S 31′50; 47°EO 01′42), and Tsarahonenana (Site H, 18°S 33′30; 47°EO 01′49) were sampled in March 2014 (Fig. 1). In April 2013, human landing catches (HLC), Centers for Disease Control and Prevention simple light traps (CDC LT), collection of mosquitoes resting indoors (MRI) and collection of mosquito resting in stables by oral aspirator were carried out. In May 2013 and March 2014, collection of mosquitoes resting in stables and HLC were carried out, respectively.

### Human landing catches

HLC were performed over two consecutive nights from the local population from 18:00 to 08:00 h in four different houses with two adult volunteers per house: one located inside and another one outside [11]. Mosquitoes coming to bite the collectors were detected using a flashlight, collected with glass tubes and placed in the collecting bags every hour. The four houses were chosen randomly in the village with no repetition. The capturers took one tablet of doxycycline over 5 days as prophylaxis against infection with the malaria parasite. The HLC were approved by the local health authority.

### Centers for Disease Control and Prevention simple light traps

CDC LT is a system that incorporates a mini-light source attracting mosquitoes, which are drawn in through the top of the trap and forced downwards by the fan into the collection bag-net. Light traps were powered by 6 V batteries. Six CDC LT were set before sunset (18:00 h) and off after sunrise (06:00 h). The CDC LT was set up outdoors over two successive nights.

### Collection of mosquitoes resting indoors

A spraying of non-remanent insecticide was performed in five houses chosen randomly where no HLC and CDC LT were made. Doors and windows were closed and eaves, which allow mosquitoes to escape from houses, were covered during the spraying. After 15 min, all dead and paralyzed mosquitoes fell onto sheets placed on the floor and over the beds and furniture before the spraying. All mosquitoes were collected and identified. Those identified as anophelines were preserved for further analysis. MRI was carried out between 06:00 and 08:00 h over 2 days.

### Collection of mosquito resting in stables

On the first and second day, two entomologists went into stables and caught mosquitoes with oral aspirators. Two stables were sampled during two mornings between 07:00 and 09:00 h.

### Identification

Mosquito identification was performed with the aid of a binocular microscope following the morphological keys [12] and (Fontenille, personal communication). Identifications were carried out in the field. Legs or wings of mosquitoes from the *An. gambiae* complex were used for PCR identification [13]. The amplification was done under the following conditions: 5 min at 94 °C followed by 30 cycles of 1 min at 94 °C, 50 s at 50 °C and 50 s at 72 °C, with a final elongation step (5 min at 72 °C). The sizes of the fragments obtained were, respectively, 315, 390 and 464 base pairs for *An. arabiensis*, *An. gambiae s.s.* and *An. merus*.

### Estimation of the entomological indices

Detection of *Plasmodium* in mosquitoes was carried out with head and thorax of all female anopheline species by ELISA CSP screening. Any sample positive following ELISA CSP screening were tested in monospecific ELISA CSP (for identification of *P. falciparum*, *Plasmodium vivax*) [14] and in PCR (for identification of *P. falciparum*, *Plasmodium malariae*, *P. vivax*, or *Plasmodium ovale*) [15]. To avoid false positive, lysates were not heated as previously described [16, 17] but all specimens were confirmed in PCR. To avoid contamination during the grinding, each specimen was put in a 1.5-ml Epperdorff with 3 ml of steel beads. Then, they were ground in Tissulyser (Tissulyser II, Qiagen^®^).

Human biting rate (HBR) was estimated for *An. funestus*, *An. mascarensis*, *An. arabiensis,* and *An. coustani*. For the sample positive to *Plasmodium*, the entomological infection rate (EIR) and the sporozoite indices were estimated. HBR is the number of bite for a given vector per person per night (bpn). The EIR is the product of the HBR, the number of bites per person per month by vector mosquitoes and the fraction of vector mosquitoes that are infectious (the sporozoite rate). The sporozoite index indicates the proportion of individual positive with *Plasmodium* among the total individuals caught for one species. These indices were calculated based on the HLC in April 2013 and March 2014.

### Blood meal analyses

Blood meal analyses were performed on blood fed females caught in stables in May 2013. Direct ELISA as previously described using antihost (IgG) conjugate against human, cow, chicken, and pig proteins were carried out [18].

## Results

### Trapping

A total of 8549 mosquitoes representing five genera and 23 species were caught (Table 1). The most abundant mosquito species were *An. coustani* (n = 3867; 45.22 %), *An. squamosus/cydippis* (n = 1284; 15.02 %), *An. mascarensis* (n = 1240; 14.50 %), *An. funestus* (n = 542; 6.34 %), and *An. arabiensis* (n = 408, 4.77 %) (Table 1).

**Table 1:** Total number and relative abundance of each species captured during the study

Species	Marondry April 2013				Marondry May 2013	Marondry March 2014	Kiangara March 2014	Total number (RA %)
	Stable	HLC	MRI	CDC LT	Stables	HLC	HLC	
*An. coustani*	*9*	*835*	*1*	*732*	*21*	*1856*	*412*	*3866 (45.22)*
*An. squamosus/cydippis*	8	102	0	195	49	520	410	1284 (15.02)
*An. mascarensis*	*14*	*28*	*0*	*105*	*531*	*251*	*311*	*1240 (14.50)*
*An. funestus*	*34*	*41*	*3*	*10*	*239*	*195*	*20*	*542 (6.34)*
*An. arabiensis*	*83*	*39*	*1*	*4*	*99*	*37*	*145*	*408 (4.77)*
*Cx. quinquefasciatus*	0	0	0	3	0	49	308	360 (4.21)
*Cx. antennatus*	0	12	0	35	13	115	100	275 (3.22)
*Ma. uniformis*	0	2	0	0	1	42	73	118 (1.38)
*An. rufipes*	0	2	0	61	3	3	43	112 (1.31)
*Cx. giganteus*	0	3	0	4	0	51	54	112 (1.31)
*An. pretoriensis*	0	0	0	0	0	0	71	71 (0.83)
*An. maculipalpis*	0	3	0	27	2	0	19	51 (0.60)
*Cx. poïcilipes*	0	0	0	0	0	30	0	30 (0.35)
*Cx. univittatus*	0	0	0	8	0	15	7	30 (0.35)
*Ae. tiptoni*	0	0	0	0	0	4	11	15 (0.18)
*Cx. decens*	0	0	0	0	0	4	6	14 (0.16)
Other species	0	2	0	5	0	7	11	23 (0.27)
Total number	148	1069	5	1189	958	3179	2001	8549

*Stables* collection of mosquitoes in stables by oral aspirator, *MRI* collection of mosquitoes resting indoors after spraying of non-remanent insecticide, *CDC LT* Centers for Disease Control and Prevention simple light trap placed indoors and outdoors, *HLC* human landing catch indoors and outdoors, *RA* relative abundance. *An., Cx., Ae., Ma.* respectively for *Anopheles, Culex, Aedes, Mansonia.* Other species include *An. flavicosta, Cx. bitaeniorhynchus, Ae. albopictus, Ae. argenteopunctatus, Ae. fowleri* and *Coquellittidia grandidieri*. Italics face indicates that malaria vectors are among the abundant species

**Fig. 2:**
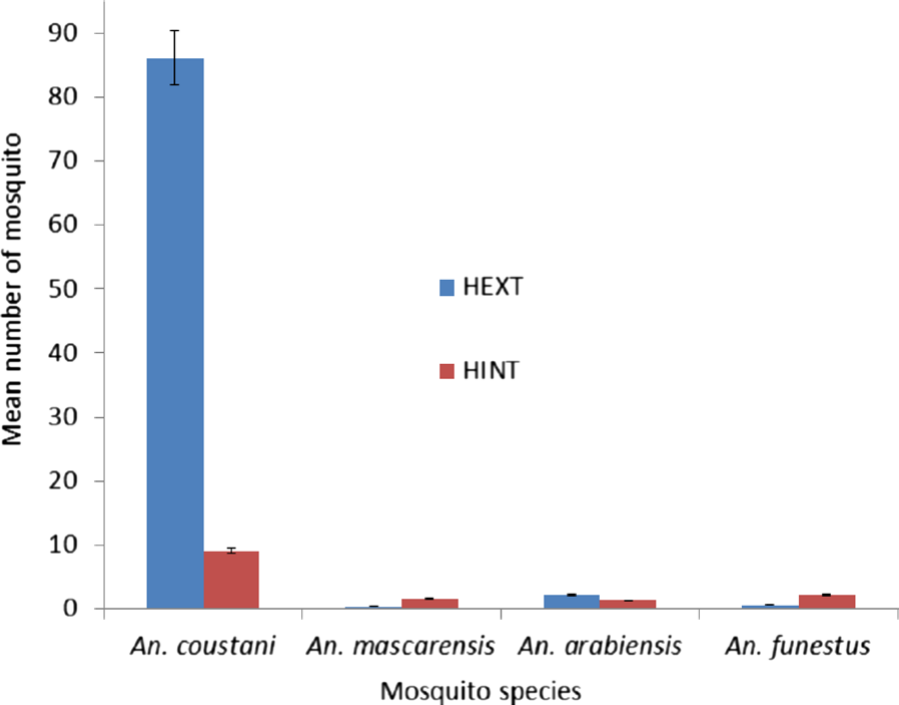
Mean number of mosquitoes captured in April 2013 by human landing catch per vector indoors and outdoors. HEXT: mean number of mosquitoes captured outdoors; HINT: mean number of mosquitoes captured indoors. These captures were made in four houses over two nights. *Vertical bars* indicate the two values of standard deviation. Comparing the trophic behaviour, *An. funestus* and *An. mascarensis* had endophagous behaviour while *An. coustani* and *An. arabiensis* were endo-exophagous

In April 2013, 2413 mosquitoes belonging to 15 species were caught during the two consecutive nights with eight *Anopheles*, six *Culex* and one *Mansonia* species. The most abundant species was *An. coustani* (n = 1578; 69.79 %) (Table 1). Comparing the trophic behaviour, *An. funestus* and *An. mascarensis* had endophagous behaviour while *An. coustani* and *An. arabiensis* were endo-exophagous (Fig. 2). One *An. arabiensis*, three *An. funestus* and one *An. coustani* were caught with MRI. Overall, vectors were more abundant in stables compared to indoors (Table 1).

Following the results in April 2013, the stables were specifically targeted for sampling in May 2013. During 2 days, nine species were caught, representing a total of 958 mosquitoes including 869 primary malaria vectors: *An. mascarensis* (n = 531, 55.54 %), *An. funestus* (n = 239, 25.00 %) and *An. arabiensis* (n = 99, 10.36 %); 21 *An. coustani* and 49 *An. squamosus/cydippis* specimens were caught in stables (Table 1). Comparing the three *An. funestus* caught with MRI during the first mission, the question arises regarding the endophily of this species.

**Fig. 3:**
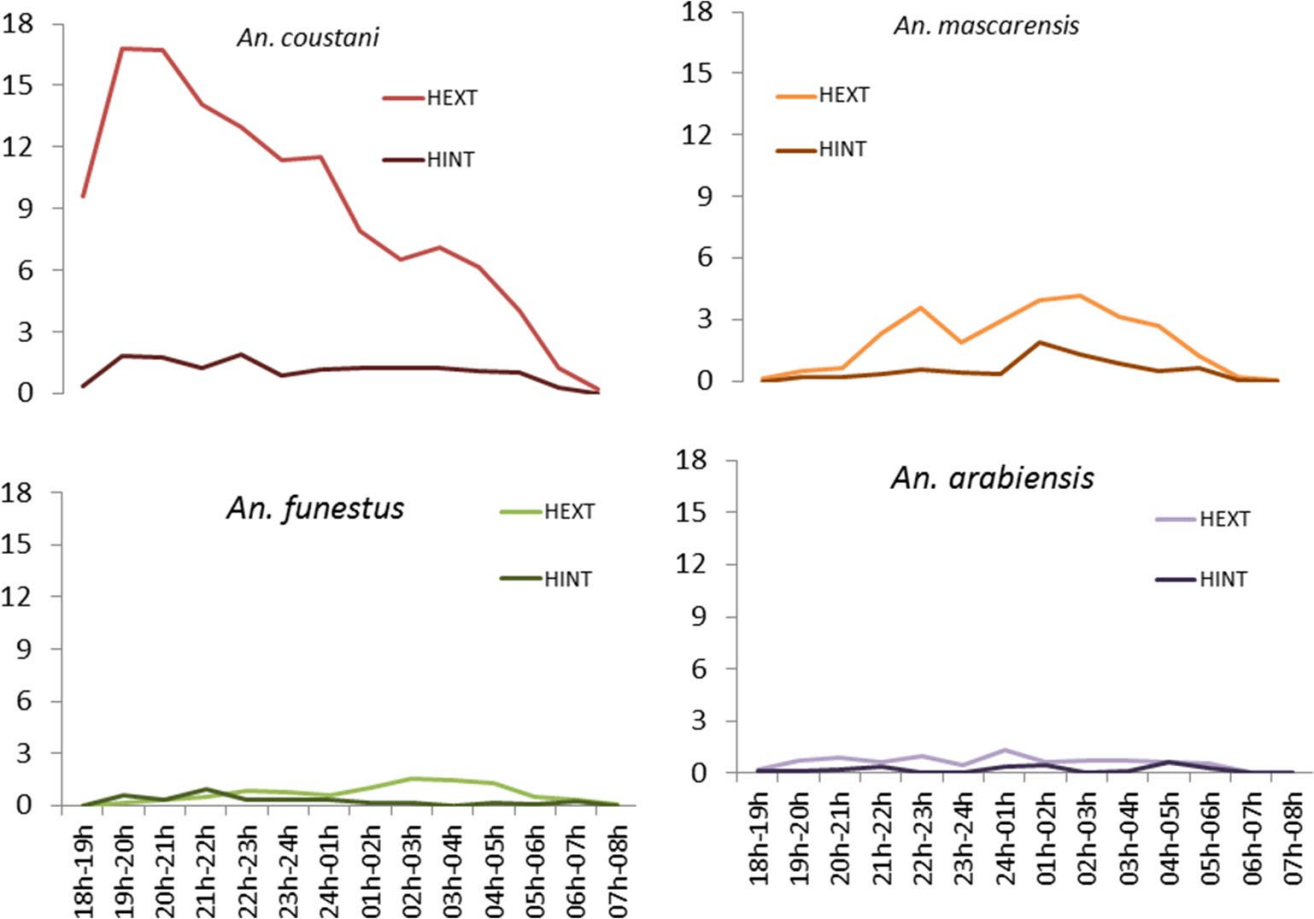
Mean number of mosquitoes captured in March 2014 by human landing catch per species indoors and outdoors. HEXT: mean number of mosquitoes captured outdoors; HINT: mean number of mosquitoes captured indoors. These captures were made in four houses over two nights in eight villages. For *An. coustani*, 38.05 % bite on humans from 18:00 to 21:00 and 05:00 to 08:00 h. Humans are not under treated mosquito nets during these times. *Anopheles mascarensis*, *An. arabiensis* and *An. funestus* had the standard aggressiveness over the night. Comparing the trophic behaviour, *An. coustani, An. arabiensis* and *An. mascarensis* had exophagous behaviour while *An. funestus* had endophagous from 18:00 to 22:00 h and exophagous from 23:00 to 07:00 h

In March 2014, 5180 mosquitoes belonging to 22 species were caught over the two consecutive nights with nine *Anopheles*, seven *Culex*, four *Aedes*, one *Mansonia*, and one *Coquillettidia* species. The most abundant species was *An. coustani* (n = 2268, 43.78 %) (Table 1). For *An. coustani*, the aggressive biting activity began earlier, i.e., before 18:00 h; 38.05 % of *An. coustani* bit humans between 18:00 and 21:00 h and from 05:00 to 08:00 h. In term of human frequency contact, humans are exposed to *An. coustani* because they are not under treated mosquito nets during these times. *Anopheles mascarensis*, *An. arabiensis* and *An. funestus* had the standard aggressiveness over the night (Fig. 3). Comparing the trophic behaviour, *An. coustani, An. arabiensis* and *An. mascarensis* had exophagous behaviour while *An. funestus* was endophagous from 18:00 to 22:00 h and exophagous from 23:00 to 07:00 h (Fig. 3).

### Entomological indices

In April 2013, one sample among the 253 primary vectors, *An. funestus*, was confirmed to be infected with *P. falciparum*. This specimen was captured indoors (Site I): the EIR was 0.21 infectious bite/person/month (ipm) (Table 2). The sporozoite index indoors for this species was 0.03 (i.e., 3 % of *An. funestus* caught were sporozoite positive).

**Table 2:** Human biting rate (HBR), sporozoite indices (SI) and entomological infection rate (EIR) for each vector in each site

Sites	Entomological indices	*An. arabiensis*		*An. coustani*		*An. funestus*		*An. mascarensis*	
		HEXT	HINT	HEXT	HINT	HEXT	HINT	HEXT	HINT
Site A	No	45	23	*133*	28	0	2	90	62
	HBR	4.50	2.30	*13.30*	2.80	0	0.20	9.00	6.20
	SI	–	–	–	0.04	–	–	–	–
	EIR	–	–	–	0.11	–	–	–	–
Site B	No	10	4	*51*	15	3	3	43	19
	HBR	1.00	0.40	*5.10*	1.50	0.30	0.30	4.30	1.90
Site C	No	49	10	*84*	39	5	2	70	14
	HBR	4.90	1.00	*8.40*	3.90	0.50	0.20	7.00	1.40
Site D	No	2	2	*57*	5	4	1	12	1
	HBR	0.20	0.20	*5.70*	0.50	0.40	0.10	1.20	0.10
Site E	No	1	2	*363*	34	98	36	71	13
	HBR	0.10	0.20	*36.30*	3.40	9.80	3.60	7.10	1.30
Site F	No	26	3	*461*	10	27	1	33	0
	HBR	2.60	0.30	*46.10*	1.00	2.70	0.10	3.30	0
	SI	–	–	0.01	–	–	–	–	–
	EIR	–	–	0.01	–	–	–	–	–
Site G	No	4	1	*505*	95	9	14	37	4
	HBR	0.40	0.10	*50.50*	9.50	0.90	1.40	3.70	0.40
Site H	No	0	0	*368*	20	8	2	85	8
	HBR	0	0	*36.80*	2.00	0.80	0.20	8.50	0.80
	SI	–	–	0.01	0.05	–	–	–	–
	EIR	–	–	0,01	–	–	–	–	–
Site I	No	18	21	*689*	146	5	36	3	25
	HBR	2.25	2.63	*86.13*	18.25	0.63	4.50	0.38	3.13
	SI	–	–	–	–	–	0.03	–	–
	EIR	–	–	–	–	–	0.21	–	–

*HBR* human biting rate, the number of bite per person per night; *SI* sporozoite indices, the proportion of individual positive with *Plasmodium* among the total individuals caught for one species, *EIR* entomological infection rate: the number of infecting bite per person per site per month. In April 2013, one sample among the 253 primary vectors, *An. funestus*, was confirmed to be infected with *P. falciparum*. This specimen was captured indoors (Site I). In March 2014, no primary vector was positive but four *An. coustani* were positive: two were positive with *P. vivax* and two with *P. falciparum*. These specimens were captured indoors (Site A) and outdoors (Sites F, H). Italics face indicates that HBR is very high for *An. coustani* mainly outdoors

In May 2013, two *An. coustani* (representing a sporozoite index of 9.5 % of all caught *An. coustani*) were confirmed to be infected with *P. vivax.* Among the 869 primary vectors, one *An. mascarensis* (0.18 % of *An. mascarensis*) was positive with *P. vivax*.

In March 2014, no primary vector was positive but four *An. coustani* were positive with *Plasmodium*. Two were positive with *P. vivax* and two with *P. falciparum*. These specimens were captured indoors (Site A) and outdoors (Sites F, H). The EIR was 0.11 ipm indoors (Site A) and 0.01 ipm outdoors (for each Sites F, H) (Table 2).

The HBR values of each site for each vector were presented in Table 2. It was higher outdoors than indoors and varied from site to site. For *An. coustani*, the value of HBR can reach up to 86.13 bpn outdoors and 18.25 bpn indoors (Site I). For the primary vector, it varied from 0 bpn indoors to 9.00 bpn outdoors for *An. mascarensis,* from 0 to 4.9 bpn for *An. arabiensis* and 0–4.50 bpn for *An. funestus.*

### Blood meal analyses

In stables, all caught mosquitoes were blood fed females (n = 958/958). The anthropophily rate was only 7.90 % (n = 39/494). This anthropophilic rate was 0.96 % (n = 2/21) for *An. coustani*. Among the main vectors, *An. funestus* regarded as an anthropophilic vector, had only 8.30 % of human blood, 7.90 % for *An*. *mascarensis* and 6.90 % for *An. arabiensis*. The other blood meal source detected was from cow blood. No blood meals from pig or from chicken were identified.

## Discussion

Six *An. coustani* females were found positive with *P. vivax* and *P. falciparum* in the field. This isolation of pathogens from wild-caught mosquitoes is one criterion to state a given species as a potential vector [19]. With the high density of *An. coustani* during entomological investigations, this species could play a role in the maintenance of *Plasmodium* transmission during the rainy season, even during the dry season. The public health importance of *An. coustani* has not previously been described. In 1988, *An. coustani* and *An. squamosus/cydippis* were suspected to transmit malaria in CHM due to their abundance [5]. *Anopheles coustani* was reported in high numbers in all regions of Madagascar up to 1900 m asl [12, 20]. The involvement of *An. coustani* in malaria transmission was also suspected in other countries. In Zambia, these mosquitoes were suspected of being a potential secondary vector according to the large proportion of caught mosquitoes anophelines coupled with an anthropophilic behaviour [21].

Ankazobe is definitively an area favourable to *Anopheles* species in the CHM. Importantly, the three main vectors were present together with significant densities at the end of the rainy season in stables, which are non-insecticide-treated areas. The number of vectors, including *An. coustani*, was very important during this study despite the low sampling effort. Even considering a low anthropophilic index for *An. coustani*, this potential vector was detected with human blood, reflecting human/vector contact. Moreover, the high level of HBR mainly outdoors for *An. coustani*, compared to the primary vector, supports contact of this vector with humans. In addition, CDC LT captured this species, suggesting that the CDC LT could be an alternative for HLC.

Vector control using IRS and insecticide-treated bed nets is the key strategy to protect humans from mosquito bites in Madagascar. In response, changes in trophic behaviour of vector species was reported [22, 23]. The bite could have occurred in the evenings or mornings when people are active outdoors and unprotected [24]. To evade insecticide, vectors could also change their feeding times, their home visit time and resting places [25]. The definition of endophilic and exophilic became ambiguous because if these terms were related to humans, mosquitoes captured in stables were exophilic. If not, all mosquitoes captured in stables were endophilic and zoo-endophagic. In Madagascar, stables are not insecticide-treated and are often attached to houses. Mosquitoes can bite in houses and exit rapidly for resting in stables. This phenomenon reduces contact with insecticide and its impact [25]. This trophic behaviour change requires a new strategy in vector control because stables must be treated. A slight change of *An. funestus* behaviour was detected ten years ago in Madagascar with the hypothesis of a change from human to animal biting [26]. This feeding preference change was observed during this study with less than 10 % of human blood-engorged females.

## Conclusion

During this study, *An. coustani* species were positive with *P. falciparum* and *P. vivax.* The very high abundance and detection of *Plasmodium* coupled with opportunistic feeding behaviour in villages with malaria cases supports its role in malaria transmission in Madagascar. Regarding insecticide treatment against the vectors, not only houses but also stables, as major resting sites of *Anopheles* vector species, need to be treated as priority. The current and rapid changes of behaviour of the main vectors and the implication of *An. coustani* as a new vector require an adaptation of control methods.

## Abbreviations

Asl: above sea level
bpn: bite/person/night
CDC: Centers for Disease Control and Prevention
CHM: Central Highlands of Madagascar
CSP: circumsporozoite protein
DDT: dichlorodiphenyltrichloroethane
EIR: entomological infection rate
ELISA: enzyme-linked immunosorbent assay
HBR: human biting rate
HLC: human landing catch
ipm: infectious bite/person/month
IRS: indoor residual spraying
LLIN: long-lasting insecticidal nets
LT: light trap
MRI: mosquitoes resting indoors
PCR: polymerase chain reaction
SI: sporozoite indices
